# Prevalence of distress, its associated factors and referral to support services in people with cancer

**DOI:** 10.1111/jocn.15794

**Published:** 2021-05-04

**Authors:** Deborah Kirk, Istvan Kabdebo, Lisa Whitehead

**Affiliations:** ^1^ School of Nursing and Midwifery Edith Cowan University Joondalup WA Australia

**Keywords:** Australia, cancer survivors, cross‐sectional studies, logistic models, oncology nursing, psychological distress, referral and consultation

## Abstract

**Aims and objectives:**

To (i) characterise prevalence of distress amongst people diagnosed with cancer, (ii) determine factors associated with increasing distress, (iii) describe reported problems for those with clinically significant distress and (iv) investigate the factors associated with referral to support services.

**Background:**

International studies report a high prevalence of clinically significant distress in people with cancer. Australian studies are notably lacking. Additionally, clinicians still do not fully understand the factors associated with cancer‐related distress.

**Design:**

Period prevalence study.

**Methods:**

Distress screening data were analysed for 1,071 people accessing the Cancer Council Western Australia information and support line between 01/01/2016–31/12/2018. These data included people's demographics, cancer diagnoses, level of distress, reported problems and the service to which they were referred. Distress and reported problems were measured using the National Comprehensive Cancer Network Distress Thermometer and Problem List. A partial proportional logistic regression model was constructed to determine which factors were associated with increasing levels of distress. Standard binary logistic regression models were used to investigate factors associated with referral to support services. The STROBE checklist was followed.

**Results:**

Prevalence of clinically significant distress was high. Self‐reported depression, sadness, worry and a lack of control over treatment decisions were significantly associated with increasing distress. Emotional problems were the most prevalent problems for people with clinically significant distress. Most people were referred to emotional health services, with depression, fatigue, living regionally and higher socioeconomic status associated with referral.

**Conclusions:**

Emotional problems such as depression, sadness and worry are associated with increasing levels of distress.

**Relevance to clinical practice:**

Not all factors associated with referral to support services were those associated with increasing levels of distress. This suggests that other factors may be more influential to referral decisions.

**What does this paper contribute to the wider global clinical community?**

- More than nine in ten people accessing the Cancer Council Information and Support line reported clinically significant levels of distress.
- Emotional problems were the prevalent problems amongst those experiencing distress.
- Not all factors associated with distress were associated with referral to support services.

## 1 INTRODUCTION

It is estimated that over 145,000 new cases of cancer will be diagnosed in Australia in 2020 (Australian Institute of Health & Welfare [AIHW], 2020). Australia's population growth and ageing demographic will contribute to a growing incidence rate for all cancers over the coming decades (AIHW, 2020). Marked improvements in early detection and treatment have improved the overall survival rate of cancer, with seven in ten Australians now surviving at least five years postdiagnosis (AIHW, 2019). The World Health Organization estimates that the figure of 25 million people alive in 2008 with a prior diagnosis of cancer will triple by 2030 to 75 million cancer survivors within 5 years of diagnosis, reflecting the dramatic expected increase in people living longer postcancer diagnosis and the need for the focus of care to extend beyond treatment (International Agency for Research on Cancer [IARC], 2008). Post‐treatment, between 30%–50% of cancer survivors describe experiencing unmet needs (Knobf et al., 2012), some many years post‐treatment (Bennett et al., 2010). Research has found that higher unmet needs are associated with the following sociodemographic, clinical and psychological characteristics: younger and older age, ethnicity, intensity of treatment, cancer type, quality of life, low income, anxiety and depression (Beckjord et al., 2014; Butow et al., 2013; Kent et al., 2012; Knobf et al., 2012; Park & Hwang, 2012;).

The evolution of cancer treatment as a chronic disease has resulted in unintended psychosocial effects which impact the quality of life of many people diagnosed with cancer (Grassi et al., 2017; Phillips & Currow, 2010). Unmet needs are directly associated with distress, defined by the National Comprehensive Cancer Network as a ‘multifactorial, unpleasant experience of a psychological (cognitive, behavioural and emotional), social, spiritual and/or physical nature that may interfere with the ability to cope effectively with cancer, its physical symptoms and its treatment' (Holland et al., 2019). It has also been noted that ‘distress extends along a continuum, ranging from common feelings of vulnerability, sadness, and fears to problems that can become disabling, such as depression, anxiety, panic, social‐isolation, and existential and spiritual crisis’ (Holland et al., 2019). While a certain level of distress is to be expected in those with cancer, without adequate monitoring and early intervention, distress could lead to serious mental health issues, which are known to compound existing physical problems and impact mortality (Pinquart & Duberstein, 2010).

## 2 BACKGROUND

Distress can result from a person's initial reaction to their cancer diagnosis and may persist throughout the various stages of the disease (Hamilton et al., 2018). Distress can emerge due to uncertainty about their prognosis and linger as a result of the side‐effects of their treatment during survivorship (Hamilton et al., 2018). Demographic characteristics such as gender, age, race and social remoteness may also influence a person's level of emotional distress (Butow et al., 2012; Hamilton et al., 2018). For example, younger people, singletons and women with cancer have been identified as people with a generally high level of distress (Hamilton et al., 2018). Similarly, appetite suppression, weight loss and a change in eating habits have been linked with an individual's level of distress following a cancer diagnosis (Hopkinson et al., 2006).

Certain institutions consider emotional distress in those with cancer as the sixth vital sign in cancer care (Bultz & Carlson, 2006). Clinically significant levels of distress amongst those with cancer have been reported in numerous international studies (Carlson et al., 2010; Zabora et al., 2001). For example, approximately two thirds of people with lung cancer and half of those with breast cancer reported clinically significant distress in one Canadian study (Carlson et al., 2010). Furthermore, a recent study in Germany found psychological distress in more than half of all screened people with cancer (Mehnert et al., 2018). In that study, fatigue, sleep problems and sadness were the most prevalent problems associated with higher levels of distress (Mehnert et al., 2018). Many studies have also indicated a potential causal link between distress and poorer quality of life, which may also negatively impact survival (Brown et al., 2003; Massie, 2004; Pirl et al., 2012).

There has been limited evidence put forward in the Australian setting measuring the extent of cancer‐related distress and determining its associated risk factors. Prior research suggests that people diagnosed with cancer living in rural locations in Australia may experience higher levels of distress, likely due to difficulties associated with accessing cancer treatment and psychosocial support services (Gunn et al., 2013; Watts et al., 2016). As the burden of cancer continues to grow, so will the associated burden of distress. Therefore, it is important to provide further context to this distress and help determine whether there are differences in levels of distress across cancer groups, between genders or between age groups in an Australian population. Additionally, it is important to understand what may be influencing this distress and how it is being addressed.

It has been demonstrated that the psychosocial needs of people with cancer are not met, validated or addressed within the context of cancer care unless the issue has been raised by the cancer treatment team (Sharpe et al., 2004). To that end, time pressures and lack of knowledge regarding psychosocial needs have been documented as barriers to adequate assessment (Tavernier et al., 2013). Screening tools have been developed and have been found to be effective and feasible in reliably identifying distress and the psychosocial needs of those with cancer (Mitchell, 2007; Zabora et al., 2001). However, research has demonstrated that institutions are still not routinely performing this type of screening (Deshields et al., 2013; Zebrack et al., 2017).

In response to international guidelines calling for systematic screening for distress amongst individuals diagnosed with cancer; in 2013, cancer nurses operating on behalf of Cancer Council Western Australia (CCWA) started using a validated distress screening tool as part of their provision of support to people affected by cancer accessing the Cancer Council Information and Support line. The support line connects those affected by cancer across urban, regional and remote WA to cancer nurses who provide information, support, guidance and referral to a wide variety of services tailored to a person's needs. People accessing this service from regional and remote WA are referred to regional Cancer Support Service Coordinators (CSCs) who provide guidance on location‐specific services and resources.

Using a non‐identifiable data set extracted from the CCWA information and support line database, this study aimed to (i) characterise prevalence of clinically significant distress amongst people diagnosed with cancer, (ii) determine factors associated with increasing levels of distress, (iii) describe the most commonly reported problems for those with clinically significant levels of distress and (iv) investigate the factors associated with referral to cancer support services.

## 3 METHODS

### 3.1 Design and sample

All people with cancer who accessed the CCWA information and support line service between 01/01/2016–31/12/2018 were captured in this period prevalence study. The telephone support line offered through this service is the primary mode of engagement for people diagnosed with cancer (Cancer Council Western Australia [CCWA], 2020a), although equivalent in‐person support is offered at certain designated CCWA offices and satellite hubs across metropolitan and regional WA (CCWA, 2020c). All people accessing this service regardless of medium (phone, email and in‐person) are screened for distress by a cancer nurse or CSC who record users’ information on a client database. The information collected includes people's self‐reported distress score and problems as well as demographic and diagnosis‐related information, such as date of birth, sex, ethnicity, residential postcode, cancer diagnosis, date of diagnosis, stage of disease and whether that person had more than one type of cancer. Following distress screening, if a person is referred to a service, the service to which a person is referred is also recorded. This may include one of the many services offered by CCWA, or an external service, depending on a person's needs. These non‐identifiable data were extracted from the CCWA information and support line database by a CCWA representative and provided to the research team for the purpose of analysis. Where a person accessed the service more than once during the observation period, only their first recorded measures were used.

### 3.2 Ethical considerations

People with cancer are referred to the CCWA Information and Support Line service by numerous sources including Cancer Nurses, Allied Health professionals, General Practitioners (GPs), oncologists, hospital social workers and through self‐referral (Watts et al., 2016). At the first point of engagement with the service, the responsible CSC or Cancer Nurse explains the rationale for distress screening and obtains informed verbal consent for the collection and use of their data for the dual purposes of providing support and future research (CCWA, 2020a; Watts et al., 2016). Following the provision of consent, people are screened for distress by the CSC or Cancer Nurse and referred to the appropriate support service (if required). People with cancer accessing the service were required to be 18 or over, be proficient in English and able to provide informed consent (Watts et al., 2016). Ethical approval for this study was granted by the Human Research Ethics Committee of Edith Cowan University (#21823).

### 3.3 Instruments and measures

#### 3.3.1 Distress thermometer

A person's level of distress was measured using the Distress Thermometer (DT) developed by the National Comprehensive Cancer Network (NCCN) (National Comprehensive Cancer Network (NCCN), 2020). The DT asks people to rate their level of distress over the past week on a scale of 0 (no distress) to 10 (extreme distress). The DT is completed by the cancer nurse on behalf of the person during their screening call. A score of 4 or higher has been identified as a clinically significant indicator for distress signalling that a person requires further questioning, follow‐up or even referral to a service appropriate to their needs (NCCN, 2020). A score of 7 or higher has been identified in the literature as an indicator of ‘severe’ distress necessitating urgent intervention (Carlson et al., 2019).

#### 3.3.2 Problem list

Accompanying the DT is a checklist of problems, termed ‘The Problem List’ (PL). The PL contains 39 yes/no questions relating to the practical, physical, family, emotional and spiritual/religious concerns of the individual. Individuals are self‐report whether they are affected by any of the 39 listed problems. The PL is delivered in conjunction with the DT as it serves to inform those screening the person of the potential source(s) of their distress (NCCN, 2020). The PL helps identify a person's key concerns across the five problem domains and is used as a guide by the cancer nurses and CSCs to adequately address an individual's needs and, where necessary, refer them to the appropriate service.

### 3.4 Data preparation

Based on the available data (specified in section 3.1), several transformations were made to bolster data analysis. For example, measures for socioeconomic status (SES) and accessibility to services were assigned to people using their recorded residential postcodes. SES was determined using the Socio‐Economic Indexes for Areas (SEIFA) (Australian Bureau of Statistics [ABS], 2018b). SEIFA is based on the Index of Relative Socioeconomic disadvantage (IRSD), which assigns a score to each suburb/postcode based on that suburb's accessibility to employment, education and income (ABS, 2018b). IRSD scores are grouped as deciles at the State and National level and are made publicly available by the Australian Bureau of Statistics (ABS). Deciles were transformed into quintiles and assigned to participants based on their residential postcodes. Quintiles were ordered from most to least disadvantaged (1–5).

A person's accessibility to services was determined with respect to the Accessibility/Remoteness Index of Australia (ARIA) score assigned to their residential postcodes. ARIA functions as a scored measure of a geographical area's accessibility to goods, services and opportunities for social interaction (ABS, 2020). ARIA scores are grouped according to five categories constituting highly accessible, accessible, moderately accessible, remote and very remote (ABS, 2020). They can also be considered as major cities, inner regional areas, outer regional areas, remote areas and very remote areas. The process of assigning ARIA groups to postcodes was conducted using the ARIA lookup tool developed by the Psycho‐oncology Co‐operative Research Group based at The University of Sydney (University of Sydney, 2020). Due to small numbers, people living in remote and very remote areas were combined into one ARIA category.

People's self‐reported cancer diagnosis/diagnoses are assigned an International Classification of Disease (ICD) (10th Revision) code by CCWA database representatives. Using a bottom‐up approach, thirteen distinct cancer groups were created based on those recorded ICD codes in consultation with the International Statistical Classification of Diseases and Related Health Problems 10th Revision handbook (World Health Organisation [WHO], 2016). Briefly, these cancer groups included: bone, breast, digestive, endocrine, eye/brain/central nervous system, female genital organs, leukaemia and lymphoma, male genital organs, mesothelial and soft tissue, oral, respiratory, skin and urinary cancers. The associated ICD codes for each created cancer group are listed in Appendix S1 (S1).

Cancer nurses and CSCs referred individuals to services based on their level of distress and their problems identified in the PL. In this sample, there were 123 unique services listed to which a person could be referred. These consisted of both internal CCWA‐run services providing; accommodation, complementary therapies, counselling, exercise and meditation courses, legal, financial and practical assistance, support groups (CCWA, 2020b), as well as external affiliated services. These services were grouped into one of the following categories: emotional health service, physical health service, practical service, informational service, multiple services or other service. Physical health services included GPs, hospitals and clinics. Emotional health services included counsellors, psychologists and therapists. Practical services included financial advisors, housing services and cooking and cleaning services. Informational services included cancer‐specific educational resources. Where a service was multifaceted and could not be reasonably assigned to a single category, it was grouped as a ‘multiple’ service. Where a service did not fit into either of the aforementioned categories, it was grouped as an ‘other’ service. The associated types of service for each service category are listed in Appendix S1 (S2).

A person's DT score was recorded as a discrete ordinal variable from 0–10. For the purpose of analysis, they were grouped according to the following categories: low distress (0–3), moderate distress (4–6) and severe distress (7–10). The reporting of this study's procedures followed The Strengthening the Reporting of Observational Studies in Epidemiology (STROBE) checklist (Von Elm et al., 2007), available in Appendix S1 (S3).

### 3.5 Data analysis

Prevalence of distress, sample demographics, diagnosis‐related information and the services to which people were referred were reported using descriptive statistics. The most frequently reported problems identified on the PL (identified by ≥15% of the sample) were reported graphically via bar chart, stratified according to levels of distress. Cancer diagnoses were grouped and presented both tabularly and graphically via bar chart in Appendix S1 (S4).

Demographic variables and distress screening variables (those pertaining to the DT and PL) were included in a multivariate ordinal logistic regression model to determine which factors were associated with increasing levels of distress (low, moderate and severe distress). A chi‐square score test resulted in the rejection of the proportional odds assumption in the model. The specific variables contributing to non‐proportional odds were identified visually using Mosaic plots as suggested by Downer (2018), (Appendix S1: S5–S7). Consequently, a partial proportional cumulative logit model, as described in Peterson and Harrell Jr. (1990), was fit to simultaneously account for differential and non‐differential impacts of the explanatory variables on the outcome. Likelihood ratio tests were conducted to confirm that the model which allowed for partial proportional odds did not have a significantly worse fit for the data than the proportional odds model. Collinearity and multicollinearity of variables in the model were assessed with respect to tests for the following measures: covariance, variance inflation factor and tolerance.

Multiple binary logistic regression models were constructed to determine which demographic variables and distress screening variables were associated with referral to the most commonly reported type of service (practical, informational, physical or emotional health service), that is those to which 15% or more of the total sample were referred. Each binary logistic regression model separately investigated the factors associated with referral to one type of service, where referral to that service constituted a positive outcome, and referral to any other type of service constituted a negative outcome.

Stepwise automated variable selection was used to determine which variables to include in each of the logistic regression models. Statistical significance for automated variable selection and all other statistical tests was considered with respect to an ∂ value of 0.05. Odds ratios (ORs) and their associated 95% confidence intervals (95% CIs) were presented for the partial proportion cumulative logit model and the binary logistic regression models.

## 4 RESULTS

A total of 1,071 people accessed the CCWA information and support line between 01/01/2016–31/12/2018 (Table 1). Most people accessed this service by phone (71.8%), followed by in‐person visit (26.98%) and email (1.21%). The majority (91%) reported a clinically significant level of distress (score of ≥4), and over half of the group (56%) reported severe psychosocial distress (score of ≥7). The sample was predominantly female (70%) with over half of all people (51.35%) aged between 50–69 years. The mean age of the sample was 56.77 years (standard deviation = 13.25). Approximately two thirds of those accessing the service were within one year of a cancer diagnosis. Most people accessing the service were either early stage (44.35%) or diagnosed with widespread or advanced cancer (30.63%). Few people reported a second primary diagnosis of cancer (1.59%) or being in the terminal stage of their disease (1.96%). Data relating to ethnicity were not recorded consistently. Of the data recorded, most people accessing the service were white non‐Indigenous Australian, European (predominantly British or Irish) or New Zealander. Most people reported living within or near major cities (65.08%), with approximately one third of the sample represented by regional (inner and outer regional) or remote and very remote geographic locations. The distribution of SES was bimodal, with a large group towards the upper end of the socioeconomic scale (35% in the second highest quintile) and a sizeable group (25%) in the lowest quintile. The majority of people were referred to ‘emotional health services’ (41.92%), followed by informational services (20.26%) and practical services (14.75%). Relatively few people were referred to physical health services (8.87%). Most people accessing the service were diagnosed with either breast cancer (26.24%) or digestive cancers (20.93%; Table 2).

**TABLE 1:** Sample demographics and characteristics

Sample characteristics	*n* (%)
Total	1,071 (100)
Distress Category	
Low (0–3)	97 (9.06)
Moderate (4–6)	376 (35.11)
Severe (7–10)	598 (55.84)
Years since cancer diagnosis	
<1	738 (68.91)
1–2	189 (17.65)
3–5	72 (6.72)
>5	29 (1.71)
Missing	43 (4.01)
Stage of cancer	
Early/localised	475 (44.35)
Metastasis/Widespread/Advanced	328 (30.63)
Recurring	63 (5.88)
Stable	44 (4.11)
Terminal	21 (1.96)
Second Primary	17 (1.59)
Remission	35 (3.27)
Unknown	60 (5.60)
Missing	28 (2.61)
Sex	
Female	748 (69.84)
Male	322 (30.07)
Other	1 (0.09)
Age group	
20–29	25 (2.33)
30–49	271 (25.30)
50–69	550 (51.35)
70–79	154 (14.38)
>79	32 (2.99)
Missing	39 (3.64)
Ethnicity	
Non‐Aboriginal and Torres Strait Islander	381 (35.57)
Aboriginal and Torres Strait Islander	10 (0.93)
Other	124 (11.58)
Missing	556 (51.91)
ARIA Category	
Highly accessible (major cities)	697 (65.08)
Accessible (Inner regional)	245 (22.88)
Moderately accessible (outer regional)	104 (9.71)
Remote and very remote	14 (1.31)
Missing	11 (1.03)
State SEIFA Quintile	
Q1 (most disadvantaged)	263 (24.56)
Q2	139 (12.98)
Q3	201 (18.77)
Q4	372 (34.73)
Q5 (least disadvantaged)	85 (7.94)
Missing	11 (1.03)
Person referred to	
Service providing emotional support	449 (41.92)
Informational service	217 (20.26)
Service providing practical support	158 (14.75)
Physical health service	95 (8.87)
Missing	88 (8.22)
‘Multiple’ service	62 (5.79)
Other service	2 (0.19)

Abbreviations: ARIA, Accessibility/Remoteness Index for Areas; ATSI, Aboriginal and Torres Strait Islander; Missing, Data unavailable for the specified number of people; *n*, Number; Q, Quintile; SEIFA, Socio‐economic Index for Areas.

**TABLE 2:** Cancer diagnoses by group

Cancer groups	Cancer cases (*n*)^a^	Proportion of total cancers (%)
Bone	10	0.90
Breast	292	26.24
Digestive	233	20.93
Endocrine	20	1.80
Eye, Brain, CNS	51	4.58
Female genital organs	98	8.81
Leukemia & Lymphoma	117	10.51
Male genital organs	77	6.92
Mesothelial and soft tissue	20	1.80
Oral	30	2.70
Respiratory	83	7.46
Skin	45	4.04
Urinary	37	3.32

Abbreviations: CNS, Central Nervous System; *n*, number.

^a^ The number of cancer cases exceed the number of unique individuals in the study due to some people self‐reporting multiple cancer diagnoses.

**FIGURE 1:**
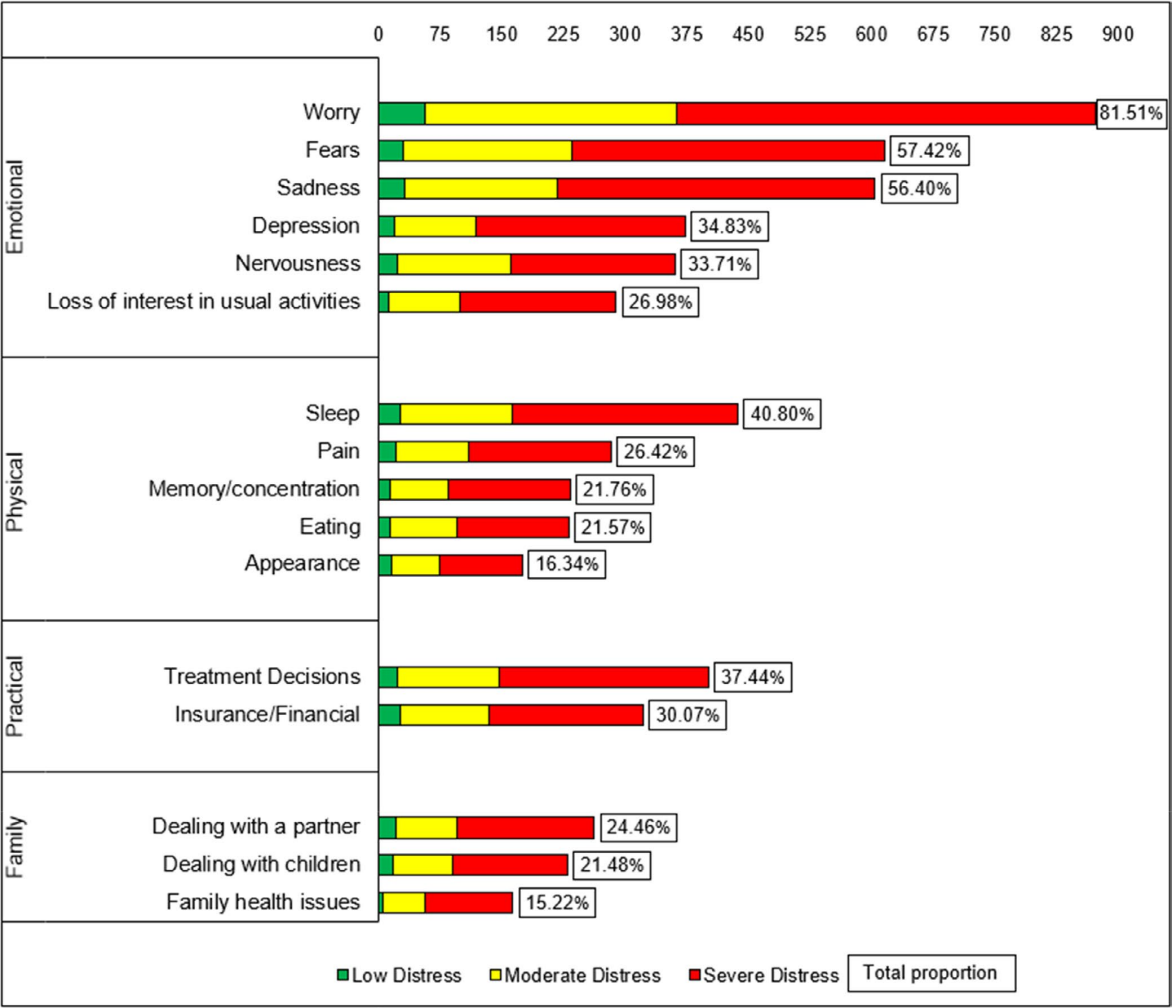
Problem list by distress category

Figure 1 represents the most commonly reported problems identified by the sample stratified by their reported level of distress. The ‘emotional problems’ grouping accounted for the three most frequently reported problems overall, with worry, fear and sadness identified as problems by 81.51%, 57.42% and 56.4% of the sample, respectively. The most frequently reported physical problem was sleep, identified by 40.8% of the sample. In the practical problem category, having concern about treatment decisions was reported by more than one third (37.44%) of all people.

Results of the partial proportional ordinal logistic regression model are shown in Table 3 and are expressed diagrammatically via forest plot in Appendix S1 (S8). The following factors significantly increased the likelihood of reporting moderate and/or severe distress: self‐reporting depression, sadness, a lack of control over treatment decisions or worry on the PL. The following factors significantly decreased the likelihood of reporting moderate and/or severe distress: being in either the 20–29 year or 70–79‐year age groups and living in either a moderately accessible (outer regional) or accessible (inner regional) area. The factors identified as correlates for increasing levels of distress showed no evidence of collinearity or multicollinearity (Appendix S1: S9 and S10).

**TABLE 3:** Factors associated with increasing levels of distress

Factors	Proportional Odds		Non–proportional odds			
	Severe vs. Moderate vs. Low Distress		Severe distress vs. Moderate distress		Moderate distress vs. low distress	
	OR	95% CIs	OR	95% CIs	OR	95% CIs
Male (female as reference)	0.77	0.58–1.01				
Depression	1.80	1.33–2.44				
Sadness	1.71	1.30–2.24				
Loss of interest in usual activities	1.36	0.99–1.86				
Lack of control over treatment decisions	1.55	1.17–2.05				
Worry			1.42	1.00–2.01	3.63	2.26–5.83
Age group (50–69 as reference)						
20–29			0.83	0.36–1.93	0.18	0.06–0.5
30–49			0.97	0.72–1.34	0.64	0.37–1.09
70–79			0.65	0.44–0.94	0.42	0.23–0.77
>79			0.87	0.41–1.83	0.51	0.15–1.77
ARIA (highly accessible as reference)						
Accessible			0.73	0.53–1.01	0.49	0.30–0.80
Moderately accessible			0.28	0.18–0.45	0.92	0.38–2.25
Remote and very remote			0.79	0.25–2.53	†	†

Abbreviations: †, Could not estimate due to low numbers; 95% Cis, 95% Confidence Intervals; ARIA, Accessibility/Remoteness Index of Australia; OR, Odds Ratio.

Several factors had proportional odds indicating that their effect on distress levels was equivalent across the levels of distress (low to moderate) and (moderate to high). For example, those who identified depression as a problem on the PL had 80% higher odds of reporting a higher level of distress relative to those who did not identify depression as a problem. Those who identified sadness as a problem had 71% higher odds of reporting a higher level of distress relative to those who did not identify sadness as a problem. Those who indicated a lack of control over treatment decisions as a problem had 55% higher odds of reporting a higher level of distress relative to those who did not identify having a lack of control over treatment decisions as a problem. Relative to being female, being male decreased the odds of reporting a higher level of distress by 23%, although this result did not reach statistical significance.

Several factors differentially affected the odds of reporting higher levels of distress. For example, those with a low level of distress who identified worry as a problem were 3.63 times more likely to report moderate distress than those who did not identify worry as a problem on the PL. However, if they were moderately distressed, they were only 42% more likely to report severe distress if they identified worry as a problem on the PL, although this result was not significant. By contrast, those in the 70–79‐year age group with a low level of distress had 58% lower odds of reporting moderate distress relative to those in the 50–69‐year age group. Similarly, those in the 70–79‐year age group with moderate distress were 35% less likely to report severe distress than those in the 50–69‐year age group. Those in the 20–29‐year age group with a low level of distress were 82% less likely to report moderate distress than those in the 50–69‐year age group. However, being 20–29 and moderately distressed was not significantly protective of reporting severe distress (95% CI = 0.36–1.93) when compared to those in the 50–69‐year age group. Relative to those living in highly accessible areas (major cities), moderately distressed people living in moderately accessible (outer regional) areas had significantly lower odds (−72%) of reporting severe distress. However, there was no significant difference in low‐distress members of these groups in terms of their likelihood of reporting moderate distress (95% CI = 0.38–2.25). Those with low levels of distress living in accessible (inner regional) areas had significantly lower odds of reporting moderate distress (−51%), relative to those living in highly accessible areas. However, this relationship was not observed for moderately distressed inner regional‐living people in terms of their odds of reporting severe distress (95% CI = 0.53–1.01) relative to those living in highly accessible areas.

Results of the binary logistic regression models are shown in Table 4. People who identified depression as a problem on the PL were 75% more likely to be referred to an emotional health service than those who did not identify depression as a problem. People who identified pain as a problem were nearly twice as likely to be referred to a practical service, relative to those who did not identify pain as a problem. People dealing with fatigue were nearly 60% more likely to be referred to an emotional health service than those who did not identify fatigue as a problem. People who identified they had financial or insurance‐related problems were more than 15 times more likely to be referred to a practical service than those who did not have financial concerns. People who were worried were more than twice as likely than those who were not to be referred to an informational service. People who self‐reported having memory or concentration problems were 81% more likely to be referred to a practical service than those who had no memory or concentration problems. People who self‐described being nervous were 83% more likely to be referred to an informational service than those who did not. People in the second highest socio‐economic quintile were 75% more likely than those in the highest quintile to be referred to an emotional health service. People living in moderately accessible (outer regional) areas were more than twice as likely to be referred to an emotional health service relative to those living in a highly accessible (metro) area.

**TABLE 4:** Factors associated with referral to each type of service

Factors	Type of service					
	Emotional		Informational		Practical	
	OR	95% CIs	OR	95% CIs	OR	95% CIs
Depression	1.75	1.29–2.37	0.54	0.36–0.83	–	–
Pain	0.60	0.43–0.83	–	–	1.98	1.28–3.09
Eating	0.67	0.47–0.98	–	–	–	–
Fatigue	1.57	1.15–2.16	0.38	0.25–0.56	–	–
Insurance/Financial	0.44	0.32–0.61	0.31	0.19–0.52	15.57	9.49–25.54
Worry	–	–	2.07	1.33–3.24	0.51	0.28–0.94
Memory/Concentration	–	–	0.47	0.25–0.87	1.81	1.13–2.89
Nervousness	–	–	1.83	1.17–2.88	–	–
Dealing with partner	–	–	0.66	0.44–0.97	–	–
Multimorbidity	–	–	–	–	4.89	0.67–35.86
SEIFA (Q5 as reference)						
Q1	1.24	0.70–2.20	–	–	0.19	0.07–0.58
Q2	1.27	0.68–2.37	–	–	0.36	0.16–0.82
Q3	0.63	0.35–1.12	–	–	0.36	0.16–0.82
Q4	1.75	1.29–2.37	–	–	1.26	0.63–2.53
ARIA (highly accessible as reference)						
Accessible	1.42	0.99–2.02	0.78	0.47–1.30	–	–
Moderately accessible	2.22	1.38–3.57	0.41	0.19–0.88	–	–
Remote and Very remote	0.55	0.14–2.14	1.85	0.48–7.06	–	–

Abbreviations: –, Factor was not identified via automated variable selection to be a significant predictor for referral to the type of service in question;95% Cis, 95% Confidence Intervals; ARIA, Accessibility/Remoteness Index; OR, Odds Ratio; Q, Quintile; SEIFA, Socio–economic Index for Areas.

## 5 DISCUSSION

### 5.1 Prevalence of distress

The first aim of this study was to characterise the prevalence of clinically significant psychosocial distress amongst a population‐based sample of people with cancer accessing a dedicated cancer information and support service. Approximately 91% of the sample reported a score of ≥4 on the DT indicating clinically significant distress, with 56% reporting severe distress (a score of ≥7). Prevalence of clinically significant distress is higher in this sample of people with cancer than all known studies using the DT. For example, previous studies have reported prevalence of clinically significant distress from 19.6% (Frost et al., 2011)–61.6% (Graves et al., 2007) and severe distress from 10% (Jacobsen et al., 2005)–20.8% (Carlson et al., 2019). In the current study, people sought out support rather than completed the measure during routine care or for the purpose of research, which may account for the higher proportions of observed distress. Two related studies involving a similar helpline service also reported clinically significant distress at 64% and 74.6%, respectively (Hawkes et al., 2010; Linehan et al., 2017).

### 5.2 Associates of distress

#### 5.2.1 Problem list items

The second aim was to identify factors associated with increasing levels of distress. Depression, sadness and a lack of control over treatment decisions were all significantly associated with increasing levels of distress. Furthermore, self‐reporting worry on the PL had the largest effect on increasing the likelihood of reporting severe distress as compared to moderate distress (OR = 3.63). Given that four of the five PL items included in the model were categorised by the ‘emotional problems’ group, our results suggest that emotional problems are significantly associated with higher levels of psychological distress. These findings build upon the work of Clover et al., (2016) who showed via classification and regression tree analysis that depression and worry were highly indicative of a clinically significant level of distress. They asserted that those emotional aspects of the PL highlighted the centrality of emotion to distress (Clover et al., 2016). Comparatively, Mehnert et al.’s (2018) international study, which used monothetic analysis, determined that sadness was the most strongly associated emotional problem with distress and that sleep and fatigue were the most strongly associated physical problems with distress. Additionally, people reporting all three of those PL items had markedly higher distress scores relative to those not reporting those problems. However, across all studies, given their cross‐sectional design the direction of causality between these reported problems and distress cannot be fully disentangled.

#### 5.2.2 Gender

Our results suggest that being male is protective against higher levels of distress; (Male vs. Female OR = 0.77 (0.58–1.01)), or alternatively phrased, that women are 30% more likely to report higher levels of distress than men (1/0.77) = (OR = 1.30). This is consistent with gender‐related disparities in psychological distress reported in individual studies (Carlson et al., 2019; Hamilton et al., 2018; Linden et al., 2012; Mehnert et al., 2018), and at a national level (ABS, 2018a), which consistently show women at higher risk of distress than men.

#### 5.2.3 Geographic location

Although some of the results are non‐significant, the direction of effect is consistently below 1 for living regionally and remotely comparative to living in urban areas, as denoted by ARIA classifications. Therefore, all other things being equal, people in this sample were less likely to report a higher level of distress if they lived regionally or remotely, compared to their urban‐dwelling counterparts. This may suggest that living outside of urban areas is protective against cancer‐related distress. This would appear contrary to evidence suggesting that distance from urban areas is consistently related to poorer cancer‐related outcomes (Butow et al., 2012), typically explained by inaccessibility to services. However, these results build‐upon and confirm earlier findings put forward by Watts et al., (2016) using a sample of 441 people accessing the same CCWA service between 2013–2014. In their study, they found no evidence that increasing remoteness was associated with higher distress and also showed that fewer problems were reported by regional‐based people than those in urban areas. Watts et al., (2016) posited that targeted government‐subsidised support structures put in place for people with cancer living further away from urban areas may serve to reduce distress. The nature of the relationship between residential location and cancer‐related distress is still not well understood (Butow et al., 2012), as it greatly differs both across and between countries. We assert that this finding may be better explained through future research elucidating psychological differences between urban‐dwelling and non‐urban‐dwelling people in terms of how they cope with psychological distress.

### 5.3 Distress and reported problems

The third aim of this study was to describe the most frequently reported psychosocial problems. The five most frequently reported problems were worry (81.51%), fear (57.42%), sadness (56.50%), sleep (40.80%) and concern about treatment decisions (37.44%). Comparatively, a recent Australian study of 1,066 oncology outpatients reported fatigue (≈30%), worry (≈24%), sleep (≈21%), pain (≈19%) and tingling (14%) as their five most commonly described problems (Clover et al., 2016). Similarly, an international study of 3,724 cancer patients reported fatigue (56%), sleep problems (51%) and difficulty getting around (47%) as their most prevalent problems (Mehnert et al., 2018). Despite these studies having a reasonably comparable demographic and clinical profile to this study across age, sex and most prevalent cancer diagnoses, only problems relating to fatigue and sleep featured in the top five problems for the samples. In Clover et al., and’s (2016) study, somatic attributes such as fatigue, pain and tingling were more commonly reported, compared to fatigue, sleep, sadness, and problems getting around (Mehnert et al, 2016) and emotional problems (worries, fears and sadness) in this study. These differences may reflect the context of data collection on the types of problems reported. For example, Mehnert et al. (2018) gathered data via in‐person interview from patients accessing outpatient cancer care facilities, cancer rehabilitation clinics and acute care hospitals (Mehnert et al., 2012), while Clover et al., and’s (2016) study collected data via in‐patient consultation with an oncologist, whereas our study primarily collected data via phone (71.8% of the sample) with a Cancer Nurse or CSC, with only 26.98% of data collected in person. Untangling the nexus of context, setting, timing, mode and person involved in data collection may help to uncover the nature of people's problems and how they relate to their perceived distress.

The final aim of this study was to determine the association between reported problems and referral to support services. Most people were referred to emotional health services, which appropriately mirrored the disproportionate burden posed by emotional problems in this sample. Given the cross‐sectional design of this study, it is unknown whether referral to these services resulted in a reduction in distress for users of the CCWA information and support line service. However, prior research conducted by Carlson et al., (2010) in a sample of people with cancer has determined that referral to psychosocial services was the strongest predictor of decreased anxiety and depression at follow‐up amongst those experiencing distress. Additionally, Gunn et al., (2013) who explored referral patterns from a rural perspective have also found that psychosocial services are considered a valued part of cancer care if people know about the services offered, have adequate access and then receive the appropriate referral. Given the variety of competing problems faced by people with cancer, future research should adopt a longitudinal design to assess whether referral to psychosocial services can reduce cancer‐related distress not associated with anxiety or depression and assess its value amongst people with cancer.

### 5.4 Strengths and limitations

To our knowledge, no other studies using regression‐based statistical methods have investigated the relationship between demographic, clinical and PL factors across low, moderate and severe distress levels as categorised by the DT. Therefore, this study is unique in its attempt to elucidate how these factors may differentially influence a person's need for follow‐up or urgent intervention based on their level of distress.

The data extracted for this study did not include a person's comorbidity status, outside of comorbid cancers. Therefore, comorbidity status could not be controlled‐for in the model examining correlates of distress. It is possible, and indeed likely, that a person affected by other chronic physical or mental conditions would experience higher distress than a person without. Adjusting for these potential confounders may have attenuated the impact of some of the identified factors on level of distress.

Measures for a person's SES (SEIFA quintiles) and accessibility to services (ARIA categories) were assigned based on their recorded residential postcodes captured between 2016–2018. While SEIFA scores were assigned according to proximal census data (2016), ARIA scores were assigned based on 2011 remoteness data, made publicly available by the ABS (University of Sydney, 2020). Therefore, certain ARIA categories may have mischaracterised the availability of services at the time a person was screened. However, as all people were assigned using these data, any potential misclassification bias was non‐differential.

Numerous factors such as health systems, geographical locations and availability of services specific to Australia and/or WA limit the generalisability of some of the findings of this study. However, it establishes a baseline for future comparison and further research within Australia. Furthermore, this study contributes valuable structured analysis of the relationship between distress and the problems faced by people with cancer across the globe.

## 6 RECOMMENDATIONS AND RELEVANCE TO PRACTICE

### 6.1 Practice

Screening for distress in clinical practice and addressing psychosocial issues through appropriate referrals and follow‐up should be part of usual care in the cancer setting. Prior research has established that screening for distress can lead to more appropriate referrals and better communication between the healthcare provider and the person experiencing cancer (Carlson et al., 2012). In this study, 91% of the sample reported clinically significant distress, with 56% of all people severely distressed. While these figures are generally higher than prior research, the widespread prevalence of clinically significant distress should call clinicians to routinely screen for distress and make practical recommendations and referrals to help alleviate this problem.

### 6.2 Research

This study suggests that depression, sadness, a lack of control over treatment decisions and worry were significantly associated with increasing levels of distress; one would reasonably assume that these same problems should have been associated with referral to services targeting a reduction in distress. While our analyses showed that depression resulted in referral to emotional health services and worry to informational services, neither sadness nor a lack of control over treatment decisions was associated with referral to any of the three examined types of service (emotional, informational or practical). By contrast, other factors such as pain, fatigue, nervousness, financial problems and problems with memory were revealed as associates for referral to these services, sometimes in competing directions (Table 4). This may indicate that these problems were influencing referral decisions more than the problems associated with distress. Structured interviews with cancer nurses responsible for referral should be conducted to determine whether there is an unequal weighting for certain reported problems on the PL in determining referral decisions. This research may also benefit from supplemental interviews from those accessing the cancer information and support line service to determine whether they felt their reported problems were being adequately addressed to reduce their level of distress.

### 6.3 Policy

Policies for distress screening are well established, but not well integrated into clinical practice and systems. Additionally, variations in referral patterns, as noted in our study, suggest that a more formal approach to psychosocial intervention could be explored. While every person should be treated as an individual when making clinical decisions, consistent processes for recognising distress and implementing interventions should be applied. Therefore, evidence‐based policies should be developed and implemented in cancer clinics to establish consistent pathways for the recognition of distress and referral to appropriate services.

## 7 CONCLUSION

Emotional problems were significantly associated with increasing levels of distress. However, not all correlates for distress were also associated with referral to services intended to reduce distress. Therefore, gaps in understanding may exist between the person living with cancer and the professional responsible for referrals in terms of adequately addressing the problems associated with their distress.

## CONFLICT OF INTEREST

None Declared.

## AUTHOR CONTRIBUTIONS

All authors have made substantial contributions to the conception and design, acquisition of data, and to the analysis and interpretation of data. All authors have been involved with drafting of manuscript and revising as necessary. All authors have given final approval of the version to be published and agreed to be accountable for all aspects of the work.

## Supporting information

Supplementary Material

Supplementary Material

## Data Availability

The data that supports the findings of this study are available in the supplementary material of this article.
