# Supplementary material for: Prevalence of distress, its associated factors and referral to support services in people with cancer
Source: J Clin Nurs. 2021 May 4;30(19-20):2873–85. doi: 10.1111/jocn.15794 (PMC8453497; doi:10.1111/jocn.15794)
Supplement: Supplementary file 2 — Supplementary Material [file JOCN-30-2873-s002.docx]

**Appendix S1**

**S1: International Classification of Diseases codes and cancer groupings for patients’ recorded diagnoses**

| **ICD Code** | **Broad ICD group** | **Narrow ICD group** | **Cancer Group** |
| --- | --- | --- | --- |
| C40 | Malignant neoplasms of bone and articular cartilage | Malignant neoplasm of bone and articular cartilage of limbs | Bone |
| C41 | Malignant neoplasms of bone and articular cartilage | Malignant neoplasm of bone and articular cartilage of other and unspecified sites | Bone |
| C50 | Malignant neoplasm of breast | Malignant neoplasm of breast | Breast |
| D05 | In situ neoplasms | Carcinoma in situ of breast | Breast |
| C15 | Malignant neoplasms of digestive organs | Malignant neoplasm of oesophagus | Digestive |
| C16 | Malignant neoplasms of digestive organs | Malignant neoplasm of stomach | Digestive |
| C17 | Malignant neoplasms of digestive organs | Malignant neoplasm of small intestine | Digestive |
| C18 | Malignant neoplasms of digestive organs | Malignant neoplasm of colon | Digestive |
| C19 | Malignant neoplasms of digestive organs | Malignant neoplasm of rectosigmoid junction | Digestive |
| C20 | Malignant neoplasms of digestive organs | Malignant neoplasm of rectum | Digestive |
| C21 | Malignant neoplasms of digestive organs | Malignant neoplasm of anus and anal canal | Digestive |
| C22 | Malignant neoplasms of digestive organs | Malignant neoplasm of liver and intrahepatic bile ducts | Digestive |
| C23 | Malignant neoplasms of digestive organs | Malignant neoplasm of gallbladder | Digestive |
| C24 | Malignant neoplasms of digestive organs | Malignant neoplasm of other and unspecified parts of biliary tract | Digestive |
| C25 | Malignant neoplasms of digestive organs | Malignant neoplasm of pancreas | Digestive |
| C26 | Malignant neoplasms of digestive organs | Malignant neoplasm of other and ill-defined digestive organs | Digestive |
| C73 | Malignant neoplasms of thyroid and other endocrine glands | Malignant neoplasm of thyroid gland | Endocrine |
| C74 | Malignant neoplasms of thyroid and other endocrine glands | Malignant neoplasm of thyroid gland | Endocrine |
| C75 | Malignant neoplasms of thyroid and other endocrine glands | Malignant neoplasm of other endocrine glands and related structures | Endocrine |
| E34 | Endocrine, nutritional and metabolic diseases | Other endocrine disorders | Endocrine |
| C69 | Malignant neoplasms of eye, brain and other parts of CNS | Malignant neoplasm of eye and adnexa | Eye, brain, CNS |
| C70 | Malignant neoplasms of eye, brain and other parts of CNS | Malignant neoplasm of meninges | Eye, brain, CNS |
| C71 | Malignant neoplasms of eye, brain and other parts of CNS | Malignant neoplasm of brain | Eye, brain, CNS |
| C72 | Malignant neoplasms of eye, brain and other parts of CNS | Malignant neoplasm of spinal cord, cranial nerves and other parts of CNS | Eye, brain, CNS |
| C51 | Malignant neoplasms of female genital organs | Malignant neoplasm of vulva | Female genital organs |
| C52 | Malignant neoplasms of female genital organs | Malignant neoplasm of vagina | Female genital organs |
| C53 | Malignant neoplasms of female genital organs | Malignant neoplasm of cervix uteri | Female genital organs |
| C54 | Malignant neoplasms of female genital organs | Malignant neoplasm of corpus uteri | Female genital organs |
| C55 | Malignant neoplasms of female genital organs | Malignant neoplasm of uterus, part unspecified | Female genital organs |
| C56 | Malignant neoplasms of female genital organs | Malignant neoplasm of ovary | Female genital organs |
| C57 | Malignant neoplasm of female genital organs | Malignant neoplasm of other and unspecified female genital organs | Female genital organs |
| D06 | In situ neoplasms | Carcinoma in situ of cervix uteri | Female genital organs |
| C77 | Malignant neoplasm of other and ill-defined sites | Secondary and unspecified malignant neoplasm of lymph nodes | Leukemia & Lymphoma |
| C81 | Malignant neoplasms, stated or presumed to be primary, of lymphoid, haematopoietic and related tissue | Hodgkin lymphoma | Leukemia & Lymphoma |
| C82 | Malignant neoplasms, stated or presumed to be primary, of lymphoid, haematopoietic and related tissue | Follicular lymphoma | Leukemia & Lymphoma |
| C83 | Malignant neoplasms, stated or presumed to be primary, of lymphoid, haematopoietic and related tissue | Non-follicular lymphoma | Leukemia & Lymphoma |
| C84 | Malignant neoplasms, stated or presumed to be primary, of lymphoid, haematopoietic and related tissue | Mature T/NK-cell lymphomas | Leukemia & Lymphoma |
| C85 | Malignant neoplasms, stated or presumed to be primary, of lymphoid, haematopoietic and related tissue | Other and unspecified types of non-Hodgkin lymphoma | Leukemia & Lymphoma |
| C90 | Malignant neoplasms, stated or presumed to be primary, of lymphoid, haematopoietic and related tissue | Multiple myeloma and malignant plasma cell neoplasms | Leukemia & Lymphoma |
| C91 | Malignant neoplasms, stated or presumed to be primary, of lymphoid, haematopoietic and related tissue | Lymphoid leukaemia | Leukemia & Lymphoma |
| C91.0 | Malignant neoplasms, stated or presumed to be primary, of lymphoid, haematopoietic and related tissue | Acute lymphoblastic leukaemia | Leukemia & Lymphoma |
| C91.1 | Lymphoid leukaemia | Chronic lymphocytic leukaemia of B-cell type | Leukemia & Lymphoma |
| C92 | Malignant neoplasms, stated or presumed to be primary, of lymphoid, haematopoietic and related tissue | Myeloid leukaemia | Leukemia & Lymphoma |
| C92.0 | Malignant neoplasms, stated or presumed to be primary, of lymphoid, haematopoietic and related tissue | Acute myeloid leukaemia | Leukemia & Lymphoma |
| C93 | Malignant neoplasms, stated or presumed to be primary, of lymphoid, haematopoietic and related tissue | Monocytic leukaemia | Leukemia & Lymphoma |
| C94 | Malignant neoplasms, stated or presumed to be primary, of lymphoid, haematopoietic and related tissue | Other leukaemias of specified cell type | Leukemia & Lymphoma |
| C95 | Malignant neoplasms, stated or presumed to be primary, of lymphoid, haematopoietic and related tissue | Leukaemias of unspecified cell type | Leukemia & Lymphoma |
| C96 | Malignant neoplasms, stated or presumed to be primary, of lymphoid, haematopoietic and related tissue | Other and unspecified malignant neoplasms of lymphoid, haematopoietic and related tissue | Leukemia & Lymphoma |
| C60 | Malignant neoplasms of male genital organs | Malignant neoplasm of penis | Male genital organs |
| C61 | Malignant neoplasms of male genital organs | Malignant neoplasm of prostate | Male genital organs |
| C62 | Malignant neoplasms of male genital organs | Malignant neoplasm of testis | Male genital organs |
| C45 | Malignant neoplasms of mesothelial and soft tissue | Mesothelioma | Mesothelial and soft tissue |
| C46 | Malignant neoplasms of mesothelial and soft tissue | Kaposi sarcoma | Mesothelial and soft tissue |
| C48 | Malignant neoplasms of mesothelial and soft tissue | Malignant neoplasm of retroperitoneum and peritoneum | Mesothelial and soft tissue |
| C49 | Malignant neoplasms of mesothelial and soft tissue | Malignant neoplasm of other connective and soft tissue | Mesothelial and soft tissue |
| C00 | Malignant neoplasms of lip, oral cavity and pharynx | Malignant neoplasm of lip | Oral |
| C01 | Malignant neoplasms of lip, oral cavity and pharynx | Malignant neoplasm of base of tongue | Oral |
| C02 | Malignant neoplasms of lip, oral cavity and pharynx | Malignant neoplasm of other and unspecified parts of tongue | Oral |
| C04 | Malignant neoplasms of lip, oral cavity and pharynx | Malignant neoplasm of floor of mouth | Oral |
| C05 | Malignant neoplasms of lip, oral cavity and pharynx | Malignant neoplasm of palate | Oral |
| C06 | Malignant neoplasms of lip, oral cavity and pharynx | Malignant neoplasm of other and unspecified parts of mouth | Oral |
| C07 | Malignant neoplasms of lip, oral cavity and pharynx | Malignant neoplasm of parotid gland | Oral |
| C08 | Malignant neoplasms of lip, oral cavity and pharynx | Malignant neoplasm of other and unspecified major salivary glands | Oral |
| C09 | Malignant neoplasms of lip, oral cavity and pharynx | Malignant neoplasm of tonsil | Oral |
| C10 | Malignant neoplasms of lip, oral cavity and pharynx | Malignant neoplasm of oropharynx | Oral |
| C11 | Malignant neoplasms of lip, oral cavity and pharynx | Malignant neoplasm of nasopharynx | Oral |
| C13 | Malignant neoplasms of lip, oral cavity and pharynx | Malignant neoplasm of hypopharynx | Oral |
| C14 | Malignant neoplasms of lip, oral cavity and pharynx | Malignant neoplasm of other and ill-defined sites in the lip, oral cavity and pharynx | Oral |
| C30 | Malignant neoplasms of respiratory and intrathoracic organs | Malignant neoplasm of nasal cavity and middle ear | Respiratory |
| C32 | Malignant neoplasms of respiratory and intrathoracic organs | Malignant neoplasm of larynx | Respiratory |
| C34 | Malignant neoplasms of respiratory and intrathoracic organs | Malignant neoplasm of bronchus and lung | Respiratory |
| C43 | Melanoma and other malignant neoplasms of skin | Malignant melanoma of skin | Skin |
| C44 | Melanoma and other malignant neoplasms of skin | Other malignant neoplasms of skin | Skin |
| D03 | In situ neoplasms | Melanoma in situ | Skin |
| D04 | In situ neoplasms | Carcinoma in situ of skin | Skin |
| C64 | Malignant neoplasms of urinary tract | Malignant neoplasm of kidney, except renal pelvis | Urinary |
| C65 | Malignant neoplasms of urinary tract | Malignant neoplasm of renal pelvis | Urinary |
| C67 | Malignant neoplasms of urinary tract | Malignant neoplasm of bladder | Urinary |
| C68 | Malignant neoplasms of urinary tract | Malignant neoplasm of other and unspecified urinary organs | Urinary |

*CNS = Central Nervous System*

**S2: Categorisation of services by services type**

| **Physical** | **Emotional** | **Practical** | **Information** | **Multiple** | **Other** |
| --- | --- | --- | --- | --- | --- |
| Appearance | Counselling | Financial | Awareness | Assigned when it was not possible to group a service into one category | Reserved for services that did not align with either physical, emotional, practical or informational categories |
| Treatment | Psychology | Housing | Education |  |  |
| Hospital | Psychotherapy | Cooking | Research |  |  |
| GP |  | Cleaning | Policy |  |  |
|  |  | Handyman | Advocacy |  |  |
|  |  | Babysitting | Evidence-based practice |  |  |

**S3: STROBE Statement—Checklist of items that should be included in reports of cross-sectional studies**

|  | Item No | Recommendation | Page No |
| --- | --- | --- | --- |
| **Title and abstract** | 1 | (*a*) Indicate the study’s design with a commonly used term in the title or the abstract | ‘Manuscript V3’ P1 |
|  |  | (*b*) Provide in the abstract an informative and balanced summary of what was done and what was found | ‘Manuscript V3’P1 |
| Introduction | | | |
| Background/rationale | 2 | Explain the scientific background and rationale for the investigation being reported | ‘Manuscript V3’ P2-5 |
| Objectives | 3 | State specific objectives, including any prespecified hypotheses | ‘Manuscript V3’ P5 |
| Methods | | | |
| Study design | 4 | Present key elements of study design early in the paper | ‘Manuscript V3’ P5 |
| Setting | 5 | Describe the setting, locations, and relevant dates, including periods of recruitment, exposure, follow-up, and data collection | ‘Manuscript V3’ P5 |
| Participants | 6 | (*a*) Give the eligibility criteria, and the sources and methods of selection of participants | ‘Manuscript V3’ P6 |
| Variables | 7 | Clearly define all outcomes, exposures, predictors, potential confounders, and effect modifiers. Give diagnostic criteria, if applicable | ‘Manuscript V3’ P6-8 |
| Data sources/ measurement | 8* | For each variable of interest, give sources of data and details of methods of assessment (measurement). Describe comparability of assessment methods if there is more than one group | ‘Manuscript V3’ P6-8 |
| Bias | 9 | Describe any efforts to address potential sources of bias | ‘Manuscript V3’ P9 |
| Study size | 10 | Explain how the study size was arrived at | ‘Manuscript V3’ P5 |
| Quantitative variables | 11 | Explain how quantitative variables were handled in the analyses. If applicable, describe which groupings were chosen and why | ‘Manuscript V3’ P7-8 |
| Statistical methods | 12 | (*a*) Describe all statistical methods, including those used to control for confounding | ‘Manuscript V3’ P8-9 |
|  |  | (*b*) Describe any methods used to examine subgroups and interactions | ‘Manuscript V3’ P9 |
|  |  | (*c*) Explain how missing data were addressed | N/A |
|  |  | (*d*) If applicable, describe analytical methods taking account of sampling strategy | N/A |
|  |  | (*e*) Describe any sensitivity analyses | N/A |
| Results | | | |
| Participants | 13* | (a) Report numbers of individuals at each stage of study—eg numbers potentially eligible, examined for eligibility, confirmed eligible, included in the study, completing follow-up, and analysed | ‘Manuscript V3’ P10 |
|  |  | (b) Give reasons for non-participation at each stage | N/A |
|  |  | (c) Consider use of a flow diagram | N/A |
| Descriptive data | 14* | (a) Give characteristics of study participants (eg demographic, clinical, social) and information on exposures and potential confounders | ‘Manuscript V3’ P10 |
|  |  | (b) Indicate number of participants with missing data for each variable of interest | ‘Tables V3’- Table 1 |
| Outcome data | 15* | Report numbers of outcome events or summary measures | ‘Tables V3’- Table 1, Table 2 |
| Main results | 16 | (*a*) Give unadjusted estimates and, if applicable, confounder-adjusted estimates and their precision (eg, 95% confidence interval). Make clear which confounders were adjusted for and why they were included | ‘Tables V3’- Table 3, Table 4 |
|  |  | (*b*) Report category boundaries when continuous variables were categorized | ‘Tables V3’- Table 1 |
|  |  | (*c*) If relevant, consider translating estimates of relative risk into absolute risk for a meaningful time period | N/A |
| Other analyses | 17 | Report other analyses done—eg analyses of subgroups and interactions, and sensitivity analyses | ‘Manuscript V3’ P12, ‘Supplementary file V3’ S5-S6, S8-S9 |
| Discussion | | | |
| Key results | 18 | Summarise key results with reference to study objectives | ‘Manuscript V3’ P13-16 |
| Limitations | 19 | Discuss limitations of the study, taking into account sources of potential bias or imprecision. Discuss both direction and magnitude of any potential bias | ‘Manuscript V3’ P16-17 |
| Interpretation | 20 | Give a cautious overall interpretation of results considering objectives, limitations, multiplicity of analyses, results from similar studies, and other relevant evidence | ‘Manuscript V3’ P18 |
| Generalisability | 21 | Discuss the generalisability (external validity) of the study results | ‘Manuscript V3’ P17 |
| Other information | | | |
| Funding | 22 | Give the source of funding and the role of the funders for the present study and, if applicable, for the original study on which the present article is based | ‘Title page V3’ P1 |

**S4: Cancer diagnoses by cancer groups**


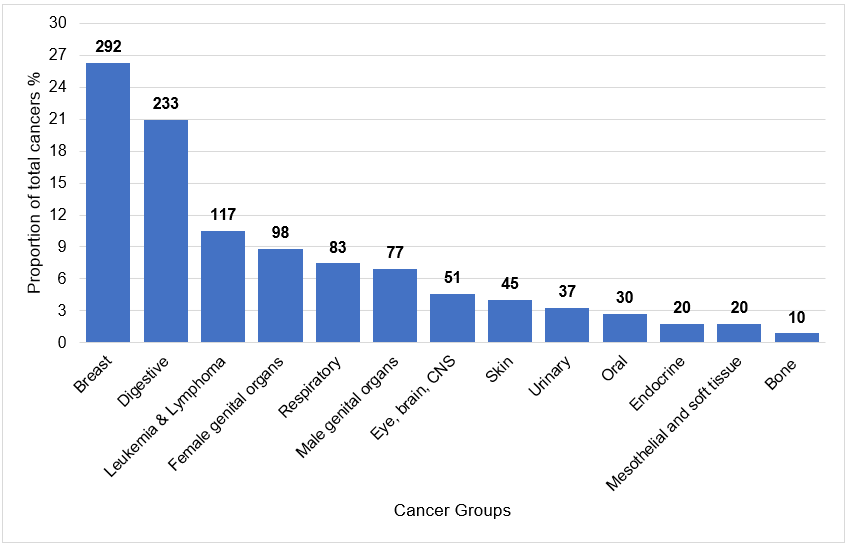


*Number of cancer cases emboldened and appended to bars*

**S5: Graphical assessment of non-proportionality of odds for ‘Worry’**

**
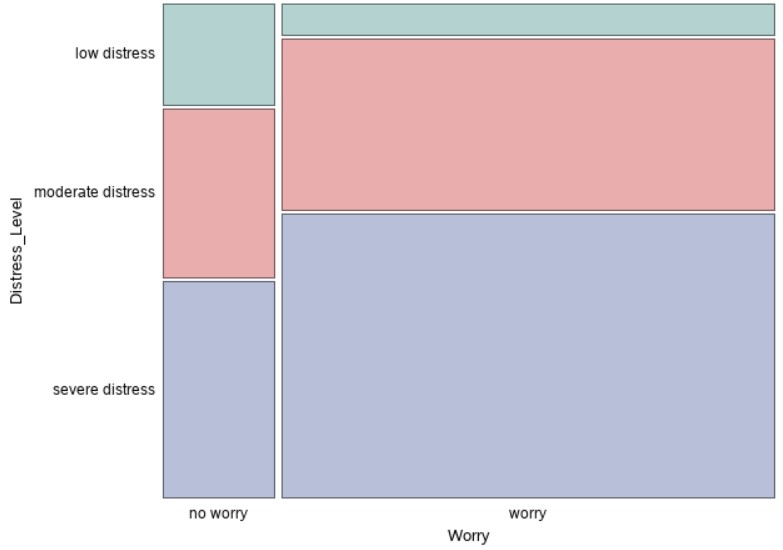
**

**S6: Graphical assessment of non-proportionality of odds for ‘Age Group’**

**
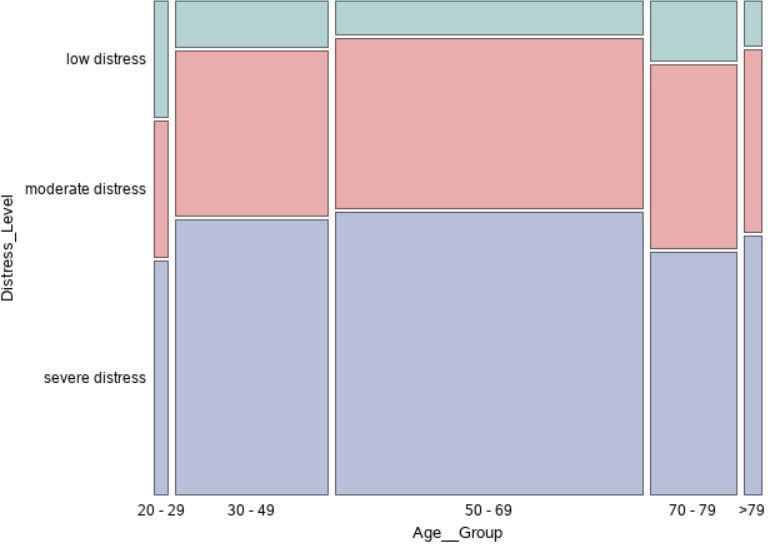
**

**S7: Graphical assessment of non-proportionality of odds for ‘ARIA Category’
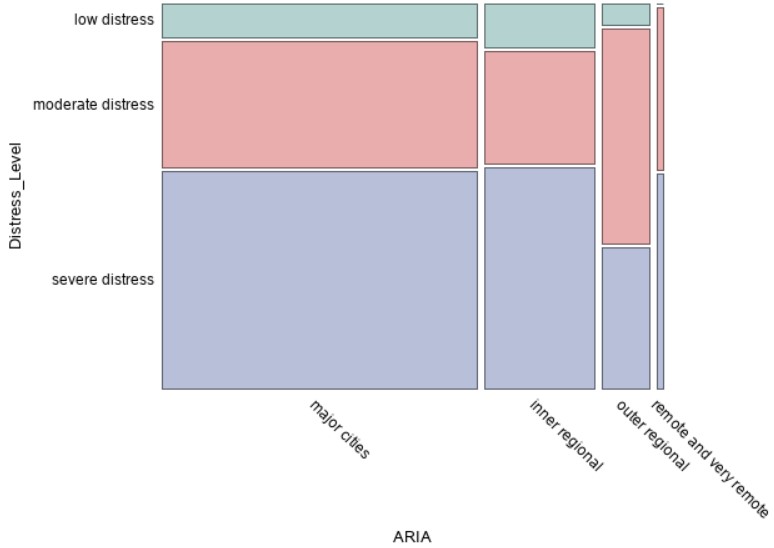
**

**S8: Forest plot of the factors associated with increasing levels of distress**

**
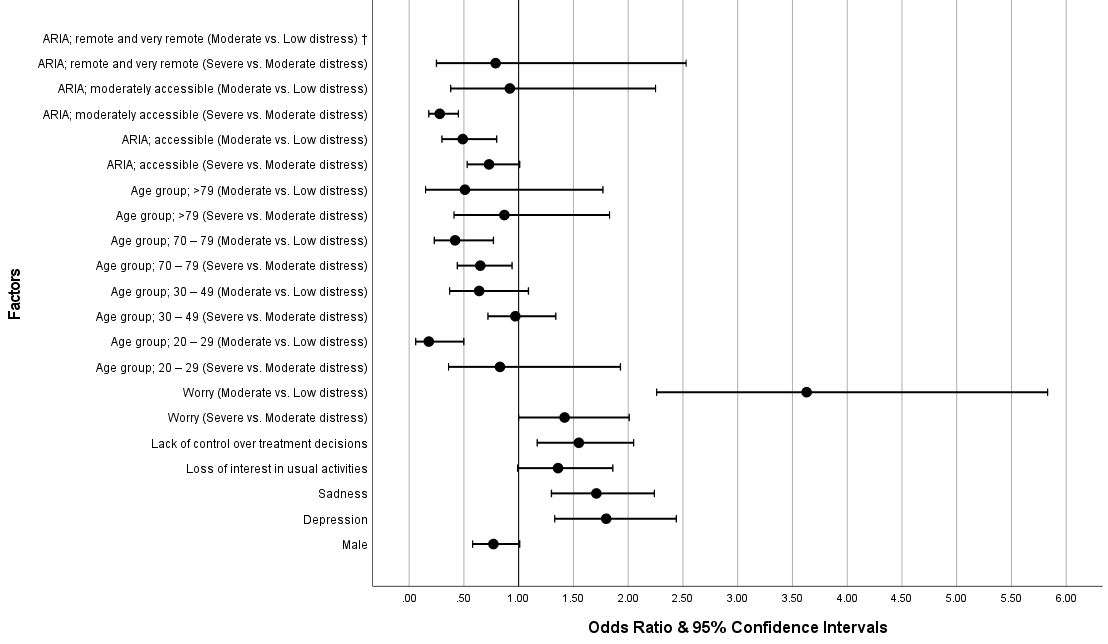
**

*† = Could not estimate due to low numbers.*

**S9: Assessment of collinearity between variables included in the model examining factors associated with increasing levels of distress**

| **Factor** | **Measure** | **Depression** | **Sadness** | **Worry** | **Age group** | **Loss of Interest in usual activities** | **Treatment decisions** | **Sex** |
| --- | --- | --- | --- | --- | --- | --- | --- | --- |
| **ARIA** | r = | 0.1 | 0.18 | 0.07 | -0.03 | 0.11 | 0.1 | -0.04 |
|  | p = | 0.001 | <.0001 | 0.02 | 0.4 | <.001 | <.001 | 0.17 |
|  | n = | 1059 | 1059 | 1059 | 1024 | 1059 | 1059 | 1059 |
| **Depression** | r = |  | 0.31 | 0.06 | 0.09 | 0.39 | 0.07 | 0.06 |
|  | p = |  | <.0001 | 0.04 | <0.01 | <.0001 | 0.02 | 0.05 |
|  | n = |  | 1070 | 1070 | 1031 | 1070 | 1070 | 1070 |
| **Sadness** | r = |  |  | 0.16 | 0.06 | 0.23 | 0.19 | -0.09 |
|  | p = |  |  | <.0001 | 0.06 | <.0001 | <.0001 | <0.01 |
|  | n = |  |  | 1070 | 1031 | 1070 | 1070 | 1070 |
| **Worry** | r = |  |  |  | < -0.01 | 0.05 | 0.23 | -0.05 |
|  | p = |  |  |  | 0.89 | 0.1 | <.0001 | 0.09 |
|  | n = |  |  |  | 1031 | 1070 | 1070 | 1070 |
| **Age group** | r = |  |  |  |  | 0.05 | 0.01 | 0.11 |
|  | p = |  |  |  |  | 0.1 | 0.83 | <.001 |
|  | n = |  |  |  |  | 1031 | 1031 | 1031 |
| **Loss of Interest in usual activities** | r = |  |  |  |  |  | 0.09 | 0.06 |
|  | p = |  |  |  |  |  | <0.01 | 0.07 |
|  | n = |  |  |  |  |  | 1070 | 1070 |
| **Treatment decisions** | r = |  |  |  |  |  |  | 0.001 |
|  | p = |  |  |  |  |  |  | 0.96 |
|  | n = |  |  |  |  |  |  | 1070 |

*r = correlation coefficient, p = p-value, n = number of participants with available data for each factor, ARIA = Accessibility/Remoteness Index of Australia.*

**S10: Assessment of multicollinearity between variables included in the model examining factors associated with increasing levels of distress**

| **Variable** | **Degrees of Freedom** | **Parameter Estimate** | **Standard Error** | **t Value** | **Pr > \|t\|** | **Tolerance** | **Variance Inflation** |
| --- | --- | --- | --- | --- | --- | --- | --- |
| **Intercept** | 1.00 | 2.24 | 0.11 | 20.69 | <.0001 | . | 0 |
| **ARIA** | 1.00 | -0.11 | 0.03 | -4.04 | <.0001 | 0.95 | 1.05 |
| **Depression** | 1.00 | 0.16 | 0.05 | 3.53 | 0.00 | 0.79 | 1.26 |
| **Sadness** | 1.00 | 0.17 | 0.04 | 3.84 | 0.00 | 0.83 | 1.21 |
| **Worry** | 1.00 | 0.22 | 0.05 | 4.15 | <.0001 | 0.93 | 1.08 |
| **Age Group** | 1.00 | -0.02 | 0.02 | -0.90 | 0.37 | 0.98 | 1.02 |
| **Loss of interest in usual activities** | 1.00 | 0.08 | 0.05 | 1.74 | 0.08 | 0.83 | 1.21 |
| **Treatment decisions** | 1.00 | 0.12 | 0.04 | 2.86 | 0.00 | 0.91 | 1.10 |
| **Sex** | 1.00 | -0.07 | 0.04 | -1.67 | 0.10 | 0.97 | 1.03 |
